# Prescribing Trends of Anticholinergic Drugs Between 1989 and 2017: A UK Biobank Study

**DOI:** 10.1093/geroni/igaa057.675

**Published:** 2020-12-16

**Authors:** Jure Mur, Simon Cox, Riccardo Marioni, Tom Russ, Graciela Muniz Terrera

**Affiliations:** 1 University of Edinburgh, Edinburgh, United Kingdom; 2 University of Edinburgh, Edinburgh, Scotland, United Kingdom; 3 University of Edinburgh, University of Edinburgh, Scotland, United Kingdom

## Abstract

Prescription drugs with anticholinergic properties are commonly prescribed and negatively impact physical performance, cognitive function, and increase the risk of falls and dementia. The prevalence of anticholinergic drugs is high in later life, when there is an increased risk of adverse drug effects. Recent, in-depth longitudinal analyses of specifically anticholinergic prescribing in Europe is lacking. Prescriptions for the UK-Biobank participants (n=222,122) were ascertained from primary care electronic patient records. We assigned anticholinergic activity to each drug by using a composite score. We used linear regression to study the association between current anticholinergic burden and time period, explore secular trends in anticholinergic use, and various demographic factors. We further explored the results in the context of different classes of prescriptions drugs. 74 distinct drugs in the sample (1.1%) had anticholinergic effects. An individual’s overall anticholinergic burden increased nonlinearly (linear estimate=0.474, quadratic estimate = 0.094, both p<2.2x10-16) between 1989 (mean=0.09, σ=0.009) and 2000 (mean=0.22, σ=0.006) and increased nonlinearly (linear estimate=0.282, quadratic estimate=0.074, both p<2.2x10-16) from 2000 to 2016 (mean=0.27, σ=0.009). The proportion of patients prescribed at least one anticholinergic drug per month increased from 6.1% to 16.7% from 1989 to 2000 and increased to 18.6% by 2016. When adjusted for sex and polypharmacy, age was negatively associated with recent cross-sectional anticholinergic burden (estimate=-0.042, p<2.2x10-16). Our results demonstrate an increase in prescribing of anticholinergic drugs over the past 30 years and indicate contemporary deprescribing of anticholinergic drugs in the later decades of life.

